# Health opportunity inequality in middle-aged and older adult cardiovascular and cerebrovascular patients

**DOI:** 10.3389/fpubh.2025.1687397

**Published:** 2026-01-12

**Authors:** Guoheng Hu, Haining Zhao, Zi Yu, Xizhao Liu

**Affiliations:** School of Public Administration, Yanshan University, Qinhuangdao, China

**Keywords:** health opportunity inequality, Shapley value decomposition, conditional inference trees, random forests, healthcare disparities

## Abstract

**Objective:**

To measure and decompose health opportunity inequality among middle-aged and older adult patients with cardiovascular and cerebrovascular diseases, providing scientific evidence for the development of targeted health policies, particularly those aimed at ensuring health equity for middle-aged and older adult individuals with cardiovascular and cerebrovascular diseases.

**Methods:**

Based on inpatient medical record data from H Province, China, we employed a pre-parameter method, combining linear regression, conditional inference trees, and random forests to measure health opportunity inequality among middle-aged and older adult patients aged 45 and above with cardiovascular and cerebrovascular diseases. The relative contributions of various environmental factors to health opportunity inequality were quantified. Additionally, unconditional quantile regression models were used to further examine the contributions of environmental factors to health opportunity inequality at different quantiles.

**Results:**

The indices of opportunity inequality for major health and overall health ranged between 7.25–12.72% and 7.13–12.54%, respectively. Key influencing factors included surgical level, the number of doctors per 1,000 people, and the number of hospital beds per 1,000 people. Furthermore, the contribution of the variance in medical expenses to opportunity inequality significantly increased with quantile values, with contributions at higher quantiles only second to the aforementioned core environmental factors.

**Conclusion:**

To promote global health equity, this study proposes several policy directions: establishing a disease–resource alignment mechanism to optimize the allocation of advanced medical resources, strengthening primary healthcare and human resource systems, reforming healthcare financing toward equitable protection, and advancing prevention-oriented, data-driven health governance.

## Introduction

With the acceleration of global population aging, the management of chronic diseases has become a core issue in public health systems worldwide. According to data from the Global Burden of Disease Collaborative Network (31), cardiovascular and cerebrovascular diseases are the leading causes of death globally, accounting for 31% of all deaths.^1^ Additionally, the disease burden shows a significant social gradient under differential resource allocation (1). As the largest developing country, China faces the paradox of “health poverty” in the process of achieving Universal Health Coverage (UHC)—the contradiction between the growth in total healthcare resources and the stagnation in health access for vulnerable groups (2), providing a unique field for testing the theory of health opportunity inequality.

Health inequality is essentially the unequal distribution of health opportunities among social members. Specifically, this inequality arises when certain groups, due to resource scarcity, disadvantaged social environments, or systemic injustice (32), are unable to equally access necessary health protections and medical services. In the public health context, “opportunity” refers not only to the formal accessibility of medical resources, but also to the set of conditions that enable individuals, within a given social structure, to avoid preventable health risks and achieve a basic level of health. This conception inherently carries a normative judgment about which health differences should be considered unjust and thus warrant policy intervention. The concept of opportunity equality was first proposed by Roemer (3), who, through his “environment-effort” dual analysis framework, categorized the unequal health outcomes of individuals into controllable effort factors and uncontrollable environmental factors, based on “individual responsibility.” Health inequalities caused by controllable effort factors are considered reasonable, while those resulting from uncontrollable environmental factors are deemed unreasonable, termed as “health opportunity inequality.” This constitutes the type of inequality that public health policies should prioritize in their interventions.

Traditionally, physiological characteristics (such as age) and socio-economic status (such as parental income and education levels) have been seen as uncontrollable environmental factors that significantly influence health opportunity inequality, making them important sources of this inequality (4–7). As research has progressed, the determinants of health opportunity inequality have expanded to include early-life experiences and the accessibility of healthcare resources. Specifically, early-life experiences often reflect the intergenerational transfer of family resources, which are typically uncontrollable environmental factors (8). Yan et al. (9) found that childhood environmental factors could explain 1 to 23% of health opportunity inequality in older age. Furthermore, the geographical distribution of healthcare resources, time costs, and transportation costs determine individuals’ access to medical services in different regions (10, 33). Rosa Dias and Jones (11), based on healthcare resource accessibility, introduced factors such as hospital density and the number of doctors per 1,000 people as environmental variables, finding that disparities in healthcare accessibility explained 32.7% of health opportunity inequality, further enriching the understanding of environmental factors in health opportunity inequality.

In terms of measuring health opportunity inequality, most studies rely on self-reported health indicators (12). While these indicators are convenient and readily available, their subjectivity may introduce biases in estimating health inequalities. To address this, some scholars have introduced objective health indicators, such as blood biomarkers, to quantify health opportunity inequality (13, 14). For example, Davillas and Jones (15) measured health opportunity inequality for chronic diseases in the UK using blood biomarkers, finding that the opportunity inequality in biomarkers explained 20% of the total inequality. In China, Ding et al. (8) analyzed pre-existing health opportunity inequality among individuals aged 60 and above using biomarkers, revealing that the opportunity inequality in biomarkers accounted for 3.75 to 29.57% of total inequality.

The existing literature provides an important foundation for understanding health opportunity inequality, but there remain gaps in the methods for measuring and decomposing this inequality. Currently, the measurement and decomposition of health opportunity inequality mainly rely on linear regression models, which present two limitations: first, linear regression assumes that the health determination equation is linear and additive, making it difficult to capture the nonlinear features and interactions among factors in health inequality; second, linear regression may overestimate the contribution of regional and provincial dummy variables to health inequality when large numbers of such variables are introduced.

To address these shortcomings, this study adopts Roemer’s opportunity equality framework and utilizes pre-parameter estimation methods. The Anderson model is used to systematically introduce multidimensional environmental variables, avoiding the limitations of a single-perspective approach. Additionally, combining conditional inference trees and random forests with traditional linear regression enables us to leverage the advantages of these machine learning methods in data processing, complex pattern recognition, and nonlinear relationship detection. This allows for the quantification and decomposition of health opportunity inequality among middle-aged and older adult patients with cardiovascular and cerebrovascular diseases. Furthermore, the study uses unconditional quantile regression models to examine the dynamic changes of health opportunity inequality at different quantiles.

The innovative contributions of this study are as follows: first, it expands the research perspective by focusing on common diseases among middle-aged and older adult populations, offering a deeper analysis of the causes of health opportunity inequality within the context of specific diseases; second, it introduces objective health indicators by using discharge diagnosis results as proxies for health, providing a more accurate reflection of individual health status and reducing subjective bias; third, it systematically incorporates environmental variables, comprehensively examining the impact of external objective conditions on health opportunity inequality within the Anderson model framework, avoiding a one-sided approach; and fourth, it innovatively integrates machine learning methods, combining conditional inference trees and random forests with traditional regression analysis to enhance the robustness of the estimates, and providing a reference for the application of machine learning techniques in this field.

## Theoretical model

### Measurement of health opportunity inequality

In Roemer's (3) “environment-effort” dual analysis framework, Fleurbaey and Peragine (16) categorize the methods for measuring opportunity inequality into “ex-ante” and “ex-post” approaches, depending on whether the “effort” variable is observed. Since the level of effort is subjective and difficult to precisely measure, the applicability of the “ex-post” approach is limited, whereas the “ex-ante” approach is more widely used. The “ex-ante” approach is further divided into parametric and non-parametric methods based on whether the income determination equation needs to be specified (17). The parametric method uses estimated coefficients of the equation and the average values of environmental variables to simulate a “counterfactual” distribution where environmental influences are removed, and then calculates the difference in inequality between the actual distribution and the “counterfactual” distribution, which represents opportunity inequality. The non-parametric method groups the sample objects based on environmental (or effort) variables and measures the inequality between different environmental groups as opportunity inequality (18). However, the non-parametric method requires a large sample size to estimate inequality. Therefore, this study mainly adopts the ex-ante parametric method for measurement.

Since all individuals within the same environmental group share the same environmental conditions, using the smoothed health status estimate of the environmental group to replace the actual health status to construct a counterfactual health distribution can, to some extent, eliminate the influence of individual effort. This study refers to the parametric construction method proposed by Ferreira and Gignoux (19), attributing the influence of effort to the environment. It is assumed that the environmental variables are exogenous, while the effort variable is further determined by environmental variables and other unobservable factors. When the outcomes achieved by all individuals depend solely on their efforts, meaning that individuals who exert the same effort receive the same return, complete opportunity equality is realized. Thus, the specific health determination equation is set as follows:


$$H_{i}=ƒ(E_{i},A_{i}(E_{i},\upsilon_{i}),\in_{i})$$ (1)

In this model, $E_{i}$ refers to environmental factors that individuals cannot control, $A_{i}$ refers to effort factors that individuals can control, and $\upsilon_{i}$ and $\in_{i}$ are error terms. Since this study only examines the impact of environmental factors on health, the above Equation 1 can be rewritten for the estimation of opportunity inequality as follows:


$$H_{i}=ƒ(E_{i},\in_{i})\;$$ (2)

We further assume that the health determination equation is linear and additive, resulting in the following equation:


$$H_{i}=\alpha E_{i}+\in_{i}$$ (3)

In this equation, α includes the direct effect of environmental factors on health, as well as the indirect effect of the environment on health through effort. $\in_{i}$ represents the error term. The Equation 3 is directly estimated using OLS to obtain ${\hat{H}}_{i}$ . Since all explanatory variables are environmental variables, the inequality index can be calculated using function I(·) to obtain the absolute value of health opportunity inequality. Following existing research, this study uses the Mean Log Deviation (MLD) as the measure of health inequality, as indicated in function see Equation 4 I(·) .


$$\theta_{\alpha }=I({\hat{H}}_{i})$$ (4)

Similarly, the actual value of health status, $H_{i}\;$ , is estimated, and the ratio with $\theta_{\alpha }$ is calculated to obtain the relative value of health opportunity inequality. This value lies between 0 and 1, as shown in the following Equation 5:


$$\theta_{r}=\frac{I({\hat{H}}_{i})}{I(H_{i})}$$ (5)

To ensure the robustness and comprehensiveness of the measurement of health opportunity inequality, this study compares three complementary modeling approaches—Linear Regression (LR), Conditional Inference Trees (CIT), and Random Forests (RF). LR serves as a parametric benchmark model assuming linear and additive relationships, while CIT and RF, as non-parametric learning methods, are capable of capturing complex, nonlinear interactions among environmental determinants that traditional models may overlook.

### Conditional inference tree

In the measurement of opportunity inequality, conditional inference trees are typically used for fitting. Unlike standard regression trees and classification trees, which use variance, accuracy, or cross-entropy as classification criteria, conditional inference trees use statistical significance tests (e.g., *p*-value-based tests) to select splitting variables. This avoids the bias issues caused by data characteristics in traditional decision trees. This study applies the conditional inference tree method to estimate Equation 2. The specific steps are as follows:

Step 1: Perform a global hypothesis test on all explanatory variables $\;E_{i}$ to examine whether the variables are statistically related to the dependent variable $H_{i}\;$ . The null hypothesis is that there is no statistical association between a given variable $E_{j}$ and $H_{i}$ see Equation 6:


$$H_{o}:E_{j}⊥H_{i}$$ (6)

If the null hypothesis for $H_{0}$ is rejected, then variable $E_{j}$ can be selected as a candidate splitting variable.

Step 2: Based on the p-value from the significance test, select the variable $E_{j}$ that is most strongly correlated with $E_{i}$ and split it.

Step 3: For the selected splitting variable $E_{j}$ , perform a significance test between sub-samples to determine the optimal splitting point (c), such that the distribution differences of health status $H_{i}$ on both sides of the split are maximized.

Step 4: Based on the optimal splitting point, recursively divide the dataset into several subgroups, and repeat the process of significance testing and selecting the optimal splitting point for each subgroup until the algorithm terminates.

### Random forest

Since a single conditional inference tree model is sensitive to sample fluctuations, even small changes in the data can lead to significant changes in the tree structure. Random forests, by aggregating multiple trees, greatly reduce the model’s sensitivity to data randomness, offering higher stability and robustness. The random forest method is used to estimate Equation 2, and the specific steps are as follows:

Step 1: Before constructing the decision trees, a two-layer randomization mechanism is used to build the model. First, a bootstrapping procedure is applied to the original sample to create B sub-sample sets, each with the same sample size. Each sub-sample is used to train a decision tree. Then, during each splitting process, m candidate variables (where m < < M, with M being the total number of variables) are randomly selected from all explanatory variables $E_{i}$ , and the best splitting variable is chosen from these.

Step 2: For each sub-sample set, random forests build fully grown decision trees (Unpruned Trees). First, at each node, the best splitting variable is selected from the randomly chosen m variables by optimizing the mean squared error (MSE) objective function. Then, the splitting process is repeated for each node until the algorithm terminates.

Step 3: Random forests generate the final model output through tree aggregation. For predicting health status, the final predicted value is the average of the predictions from all decision trees see Equation 7:


$${\hat{H}}_{i}=\frac{1}{B}\;\sum_{b=1}^{B}{\hat{H}}_{i}^{\left(b\right)}\;$$ (7)

In addition, this study uses the Grid Search Cross-Validation (Grid Search CV) method to optimize the key parameter (tree depth) of the conditional inference tree and random forest models. The parameter search range is from 1 to 20, with 5-fold cross-validation and negative mean squared error (Negative Mean Squared Error, -MSE) as the scoring criterion. The optimization results show that the optimal tree depth for different health variables is between 6 and 8. The mean squared error (MSE) and the coefficient of determination (R^2^) are calculated on the test set to evaluate the model’s predictive performance. Out-of-bag error (OOB Error) is used for unbiased estimation of model error. The results indicate that the R^2^ values of the Random Forest and Conditional Inference Tree models are significantly higher than those of the Linear Regression model (Table 1), suggesting that the linear additive structure may have limitations in capturing the complexity of health determination mechanisms. Accordingly, this study pays particular attention to the consistency and stability of opportunity inequality estimates across different methods, using multiple model cross-validation to strengthen the robustness of the conclusions.

**Table 1:** Measurement of opportunity inequality.

Health variables	(1)	(2)	(3)
	Linear Regression	Conditional Inference Trees	Random Forests
Panel A:primary health			
Absolute opportunity inequality	0.0032	0.005	0.0056
Relative opportunity inequality	0.0718	0.1147	0.1262
Total opportunity inequality	0.044	0.044	0.044
Optimal tree depth	-	6	9
MSE	1.6417	1.552	1.5287
R^2^	0.0878	0.1377	0.1506
Panel B:composite health			
Absolute opportunity inequality	0.003	0.0049	0.0052
Relative opportunity inequality	0.0703	0.1129	0.1193
Total opportunity inequality	0.0437	0.0437	0.0437
Optimal tree depth	-	6	8
MSE	1.6273	1.5417	1.5186
R^2^	0.0859	0.134	0.147

Enable approximate calculation; Use optimal tree depth.

### Decomposition of health opportunity inequality

In existing literature, scholars typically use two main methods for decomposing opportunity inequality: variance decomposition and Shapley value decomposition (20). The variance decomposition method generally relies on the assumption of feature independence or approximates interactions between features through covariance terms. However, this method has certain limitations in two key areas: first, it is difficult to accurately capture complex nonlinear interaction effects; second, it does not clearly allocate the contributions of interactions between features. Specifically, when significant interactions exist between features, variance decomposition struggles to precisely determine the contribution of each feature to these interaction effects. In contrast, the Shapley value decomposition method allows for a comprehensive allocation of interaction effects between features. This method calculates the marginal contribution of each feature in different subsets and automatically and fairly distributes interaction effects to the relevant features, thus overcoming some of the limitations of variance decomposition. Therefore, the decomposition method based on predicted means is used in this study to compute the relative contributions of each variable. However, when using the Shapley value decomposition method to precisely calculate the importance of each environmental variable, if there are k variables, it requires calculating 2^k^ permutations, with each calculation requiring retraining of the model. This leads to exponential growth in computation and high computational complexity. To address this, an approximation algorithm is employed to reduce the computational load. Specifically, this study adopts the TreeSHAP algorithm (21) implemented in the SHAP Python package to approximate the Shapley value decomposition for tree-based models such as Random Forests. TreeSHAP leverages the structure of decision trees to efficiently compute each feature’s marginal contribution through a dynamic programming process, reducing computational complexity from O(2^k^) to O(TLD^2^), where T is the number of trees, L the number of leaves, and D the tree depth. This approach preserves the theoretical consistency of Shapley values while making large-scale estimation computationally feasible. It should be noted that the TreeSHAP algorithm assumes feature independence when computing marginal contributions. The potential implications of this assumption are discussed in the limitations section.

## Variable selection and descriptive statistics

### Data source

This study is based on administrative data from the medical insurance settlement platform of H Province, China. All data were fully anonymized and de-identified before being accessed by researchers, ensuring that no personally identifiable information was included. According to the relevant provisions of the “Personal Information Protection Law of the People’s Republic of China,” the “Data Security Law of the People’s Republic of China,” and the “Ethical Review Measures for Biomedical Research Involving Human Subjects,” research using anonymized administrative data does not involve the direct recruitment of human participants and therefore does not require ethical approval or informed consent. Furthermore, this study complies with the principles of the “Declaration of Helsinki.” A stratified sampling method combined with systematic sampling was employed. After stratifying by city in H Province, patients were sorted by discharge month, and systematic sampling was conducted at equal intervals. Data from each city layer were then merged to form the final sample.

In line with the research theme, the macro data from the provincial statistical yearbook and statistical bulletins of each city in the province were matched according to city codes, resulting in an initial sample of 111,819. After age (45 and above) and disease type restrictions were applied, the final sample size was 45,470. The data include patient personal characteristics, diagnoses and treatment information, as well as medical costs, consistent with the National Health Commission’s standards for inpatient medical records. Disease types were identified and grouped using the first four digits of the ICD-10 disease codes. To avoid interference from outliers, only diseases with more than 30 cases were retained, and missing variable values were replaced with means. Additionally, a 1% winsorization was applied to the total medical expenses data.

### Variable selection

#### Health variables

In measuring health variables, the discharge diagnosis is used as the indicator. The discharge diagnosis reflects the patient’s health status at the time of discharge, taking values from 1 to 4 to represent “cured,” “improved,” “not cured,” and “death,” respectively. It is an ordinal four-category variable. To enable its use as a continuous dependent variable in subsequent model analyses, this study applies the rank-based inverse normal transformation (RINT) method to convert it into a continuous variable (22). McCaw et al. (34) noted that this method can improve model fit and effectively control Type I error.

Specifically, the procedure of the rank-based inverse normal transformation is as follows: first, the cumulative probability distribution is calculated according to the sample frequency of each discharge diagnosis category; then, the inverse cumulative distribution function of the standard normal distribution is used to map the cumulative probabilities to corresponding z-values, thereby transforming the ordinal categorical variable into an approximately continuous normal variable. This approach preserves the ordinal information of the original variable while mitigating the interpretational bias that may arise when categorical variables are directly included in regression models. The fundamental assumption of this method is that the observed ordinal outcomes are discrete manifestations of an underlying continuous health status variable. Therefore, through a monotonic mapping, they can be transformed into a continuous scale, allowing for a more accurate representation of the latent distance between different levels of health status.

The primary health indicator is measured based on the principal diagnosis at discharge. To more clearly reflect the overall health status of patients, the primary diagnosis and multiple other diagnoses are averaged to form a composite health measure. On this basis, the values for primary health and composite health are reversed, so that higher values indicate better health status.

#### Environmental variables

The Andersen model, due to its adaptability in integrating multiple environmental factors, has clear advantages in addressing health opportunity inequality. This model not only considers the effects of factors such as socio-economic status, cultural background, geographic location, and healthcare resources, but also comprehensively analyzes the interactions between these factors in influencing health opportunity inequality, rather than relying solely on socio-economic or biomedical variables. Therefore, based on the Andersen model, this study selects environmental variables from three dimensions to analyze health opportunity inequality among cardiovascular and cerebrovascular disease patients.

The first dimension is propensity factors, which primarily reflect the socio-demographic characteristics of patients. The selected variables include age, gender, ethnicity, marital status, and occupational risk. Although occupational risk is related to an individual’s choice of profession, it is typically a risk that the individual cannot fully control. The second dimension is activation-demand factors. The accessibility of healthcare resources is an important factor in reducing health inequality (10, 11, 23). This study adopts a healthcare accessibility perspective, selecting environmental variables such as the number of hospital beds per 1,000 people, the number of doctors per 1,000 people, whether the healthcare facility has a mature rail transit system, and whether the healthcare facility is located in a revolutionary old area. The third dimension is demand factors. In previous research, some scholars have argued that individuals should be responsible for their demand preferences, considering healthcare needs and preferences as rational factors contributing to healthcare outcome disparities (24, 25). However, within the framework of *Roemer's* (3) opportunity equality theory, needs and preferences are often seen as products of environmental factors. This study adopts this view, selecting variables that reflect the patient’s health status and needs, including the variance of medical expenses, which refers to the variance in medical costs for a specific diagnosis across the sample population. This measure captures the variability in healthcare utilization for patients with the same diagnosis, illustrating disparities in demand. Additionally, other variables such as surgical level, whether the patient was admitted through emergency services, and the condition at admission are also included in this analysis. Descriptive statistics are presented in Table 2.

**Table 2:** Descriptive statistics.

Variable		Mean	Standard	Min	Max
Dependent variable					
Primary health		4.99	1.329	0.472	9.207
Composite health		4.983	1.322	0.472	9.207
Environmental variables					
Propensity factors	Age	66.202	10.769	45	87
	Gender	0.502	0.5	0	1
	Ethnicity	0.971	0.169	0	1
	Marital status	0.866	0.34	0	1
	Occupational risk	0.528	0.872	0	2
Activation-demand factors	The number of hospital beds per 1,000 people	6.767	1.348	5.13	10.05
	The number of doctors per 1,000 people	2.609	0.643	1.43	3.83
	Whether the healthcare facility has a mature rail transit system	0.11	0.312	0	1
	Whether the healthcare facility is located in a revolutionary old area	0.606	0.489	0	1
Demand factors	The variance of medical costs	1.449	1.479	0	4
	Surgical level	0.789	0.874	0	2
	Whether the patient was admitted through emergency services	0.2	0.4	0	1
	The condition at admission	1.127	0.466	1	4
	N	45,470	45,470	45,470	45,470

## Results

### Measurement of health opportunity inequality

This study employs three methods—linear regression, conditional inference trees, and random forests—to measure opportunity inequality in both major and overall health outcomes. For major health, the absolute values of opportunity inequality estimated by linear regression, conditional inference trees, and random forests are 0.0032, 0.005, and 0.0056, respectively, with corresponding relative values of 7.18, 11.47, and 12.62% (see Table 1, Panel A). In terms of model fit, the random forest demonstrates the highest explanatory power, with an R^2^ of 0.1506, followed by the conditional inference tree (0.1377) and linear regression (0.0878).

For overall health, the absolute values of opportunity inequality measured by the same three methods are 0.003, 0.0049, and 0.0052, respectively, with relative values of 7.03, 11.29, and 11.93% (see Table 1, Panel B). Similarly, the random forest yields the best model fit with an R^2^ of 0.147, followed by the conditional inference tree (0.134) and linear regression (0.0859). Furthermore, for both major and overall health, the random forest produces the lowest mean squared error (MSE)—1.5287 and 1.5186, respectively—further confirming its superior performance. This may be attributed to the fact that, unlike linear regression, both conditional inference trees and random forests do not impose a pre-specified functional form, allowing them to better capture the nonlinear relationships between environmental factors and health outcomes.

### Decomposition of health opportunity inequality

To identify the relative contributions of different environmental factors to health opportunity inequality, this study applies the Shapley value decomposition method (see Table 3), based on three models: linear regression, conditional inference trees, and random forests. The results in Panel A (Major Health) show that surgical level, the number of hospital beds per 1,000 people in the treatment location, and the number of doctors per 1,000 people consistently rank among the top contributors across all three models, indicating that they are the key sources of health opportunity inequality. Specifically, surgical level has the highest contribution, ranging from 21.88% (linear regression) to 45.85% (conditional inference tree); it is followed by the number of beds per 1,000 people, contributing between 19.23% (random forest) and 21.11% (linear regression); and the number of doctors per 1,000 people, with a contribution ranging from 9.31% (random forest) to 13.87% (linear regression). Notably, in both the conditional inference tree and random forest models, the contribution of medical expenditure dispersion increases significantly, from 4.83% (conditional inference tree) to 5.74% (random forest), highlighting the impact of economic burden disparities on health opportunity inequality.

**Table 3:** Health opportunity inequality decomposition based on Shapley values.

Variables	(1)	(2)	(3)
	Linear regression	Conditional inference trees	Random forests
	Contribution (%)	Contribution (%)	Contribution (%)
Panel A:primary health			
Surgical level	21.88	45.85	40.07
The number of hospital beds per 1,000 people	21.11	21.11	19.23
The number of doctors per 1,000 people	13.87	10.5	9.31
Whether the healthcare facility has a mature rail transit system	10.04	-	-
Age	6.08	6.76	8.57
The variance of medical costs	-	4.83	5.74
Panel B:composite health			
Surgical level	21.67	46.09	42.09
The number of hospital beds per 1,000 people	21.01	20.42	19.28
The number of doctors per 1,000 people	14.15	10.58	9.88
Whether the healthcare facility has a mature rail transit system	10.01	-	-
Age	5.96	7.01	8.24
The variance of medical costs	-	4.95	5.56

The decomposition results for Panel B (Overall Health) are almost identical to those in Panel A, suggesting a high degree of robustness in the findings. Substantively, the three leading indicators—surgical level, hospital bed density, and doctor density—collectively represent a multidimensional perspective of healthcare accessibility, reflecting technical sophistication, spatial capacity, and human resource availability, respectively. This confirms the decisive role of healthcare accessibility in shaping health opportunity inequality, consistent with findings in the existing literature (11). Among these, surgical level emerges as the most influential factor, indicating that the technical complexity of medical interventions and the hierarchical distribution of resources significantly affect individuals’ health opportunities. Surgical level is closely associated with hospital technical capability, equipment conditions, and physician expertise, and can therefore serve as a proxy for the quality of care actually accessible to patients. High-level surgeries are typically concentrated in resource-rich tertiary hospitals, while lower-tier institutions tend to lack the technical capacity and resources, thus structurally limiting some patients’ access to high-quality healthcare services and exacerbating health opportunity inequality.

### Quantile differences in health opportunity inequality

To further investigate the marginal effects of environmental factors on health opportunity inequality across different health statuses, this study adopts the Unconditional Quantile Regression (UQR) method proposed by Machado and Santos Silva (35). Unlike traditional conditional quantile regression, UQR captures the structural impact of explanatory variables across the entire distribution of the health variable, thereby offering insights into distributional heterogeneity. UQR has been widely applied in the analysis of health opportunity inequality. Given that the data used are cross-sectional, no individual- or institution-level fixed effects are included to avoid over-controlling and thereby absorbing substantial variation attributable to the key explanatory variables. The model specification is consistent with that of the main analysis, employing the same set of explanatory variables. Shapley value decomposition is subsequently conducted at each quantile to identify differences in the contributions of various factors to health opportunity inequality across the health distribution.

The results reveal systematic disparities in the influence of environmental factors on both major and overall health across the distribution (see Table 4). Specifically, relative opportunity inequality in major health ranges from 5.08 to 13.49% across quantiles, while in overall health it ranges from 5 to 13.47%. Both measures exhibit a declining trend across quantiles, with relative opportunity inequality at the 25th percentile being approximately 2.69 times that at the 75th percentile. This tail effect suggests that health opportunity inequality is more pronounced among individuals with poorer health.

**Table 4:** Quantile regression-based coefficients of opportunity inequality and their decomposition.

Variables	(1)	(2)	(3)
	Q25	Q50	Q75
Panel A:primary health			
Absolute opportunity inequality	0.0058	0.0044	0.0022
Relative opportunity inequality	0.1349	0.1017	0.0508
Contribution to opportunity inequality			
The number of hospital beds per 1,000 people	13.75	20.49	21.87
The number of doctors per 1,000 people	24.84	20.81	19.23
Whether the healthcare facility has a mature rail transit system	9.31	6.87	5.67
Age	5.81	4.56	5.12
The variance of medical costs	3.15	6.05	5.98
Surgical level	16.07	21.08	23.75
Panel B:composite health			
Absolute opportunity inequality	0.0057	0.0041	0.0021
Relative opportunity inequality	0.1347	0.0974	0.05
Contribution to opportunity inequality			
The number of hospital beds per 1,000 people	13.69	20.27	21.37
The number of doctors per 1,000 people	24.82	20.93	19.7
Whether the healthcare facility has a mature rail transit system	9.4	6.82	5.84
Age	5.8	4.6	5.2
The variance of medical costs	3.11	6.32	6.34
Surgical level	16.14	21.33	24.02

The Shapley decomposition results further confirm the robustness of earlier findings: surgical level, the number of doctors per 1,000 people, and the number of hospital beds per 1,000 people remain the primary drivers of health opportunity inequality. Among these, surgical level consistently emerges as the most influential factor, with a particularly high contribution among individuals in better health. This suggests a structural pattern in which health opportunity inequality becomes more pronounced among healthier patients—that is, as overall health improves, access to advanced medical technologies and high-quality care becomes increasingly uneven. This phenomenon may stem from the interaction between health status and treatment pathways. For individuals in poorer health, medical services are typically oriented toward “necessary treatment,” with relatively fixed pathways and limited scope for choice. As such, the impact of institutional or socioeconomic differences is mitigated, resulting in a certain degree of homogeneity in access to high-level care. In contrast, patients in better health have greater flexibility in their treatment options, and their ability to access higher-quality medical services is more strongly influenced by their social capital, healthcare navigation skills, and financial capacity. Consequently, health opportunity inequality becomes more pronounced in this subgroup.

In contrast, the explanatory power of doctor density is greater for patients at the lower end of the health distribution, highlighting the critical importance of human resource accessibility for individuals in poorer health. Notably, the contribution of medical expenditure dispersion increases markedly among patients in the middle and upper quantiles. Specifically, in the case of major health, the Shapley contribution at the 50th percentile is approximately 1.92 times that at the 25th percentile; for overall health, the contribution at the 75th percentile is about 2.04 times that at the 25th percentile. This provides further support for the mechanism that links health improvement with rising health opportunity inequality. Patients in better health tend to have more discretion in choosing their care pathways, and the cost variability of services amplifies disparities in access to high-quality healthcare, thereby reinforcing structural differences in healthcare accessibility.

## Conclusion

This study utilizes inpatient medical record data from H Province, China (2019), focusing on individuals aged 45 and above diagnosed with cardiovascular and cerebrovascular diseases. It introduces two measures—major health and overall health—and employs three analytical methods: linear regression, conditional inference trees, and random forests to estimate the extent of health opportunity inequality. Additionally, it applies the Unconditional Quantile Regression (UQR) model to explore how the contributions of environmental factors to health opportunity inequality vary across different health quantiles. The findings offer important empirical evidence for promoting health equity in the context of global population aging.

The study yields two key findings. First, surgical level, the number of doctors per 1,000 residents, and the number of hospital beds per 1,000 residents in the treatment location emerge as the primary contributors to health opportunity inequality among middle-aged and older patients with cardiovascular and cerebrovascular diseases. These results highlight the persistent barriers posed by limited accessibility to healthcare resources—findings that align with international evidence, such as that from Brazil (26). Among these, surgical level consistently ranks as the most influential factor, with an especially high contribution among patients in the upper health quantiles, suggesting that access to advanced medical technologies becomes increasingly unequal as health improves. Notably, the contribution of medical expenditure dispersion is significantly higher in the better-fitting models (conditional inference trees and random forests), ranging from 4.83 to 5.74%. Furthermore, its contribution among middle-to-upper quantile groups is approximately twice that of the lower quantiles, underscoring the impact of economic burden disparities on health opportunity inequality.

Second, the analysis reveals clear quantile heterogeneity in health opportunity inequality. The relative inequality values for major and overall health range from 7.18 to 12.62% and from 7.03 to 11.93%, respectively, with a pronounced downward trend across quantiles. The value at the 25th percentile (Q25) is approximately 2.5 times that at the 75th percentile (Q75), demonstrating a tail effect. Patients in the lower quantiles, potentially due to greater clinical complexity and treatment sensitivity, are more constrained by environmental factors such as surgical level and healthcare resource density. These findings provide empirical support for the health gradient theory (27) in a clinical context, confirming that social and environmental determinants exert stronger effects on those in poorer health.

It is noteworthy that the estimated 7–12% health opportunity inequality indicates that approximately one-tenth of the total variation in patients’ health outcomes can be attributed to structural, non-effort-based factors such as access to medical resources, socioeconomic status, and healthcare affordability. In practical terms, this inequality reflects substantial disparities in recovery prospects and financial security. According to the 2025 reports by China’s National Health Commission and the Chinese Academy of Social Sciences, more than 40 million households nationwide have fallen into poverty due to illness; the rate of returning to poverty because of disease among rural families has reached 37%, with an average medical debt exceeding 80,000 yuan per household. Among urban low- and middle-income families, 42% have been forced to mortgage their homes or take high-interest loans to cope with major illnesses. These findings suggest that the level of health opportunity inequality identified in this study embodies profound structural barriers that simultaneously shape patients’ health outcomes and economic vulnerability.

Based on the measurement and decomposition of health opportunity inequality among middle-aged and older patients with cardiovascular and cerebrovascular diseases, this study identifies the critical roles of healthcare resource accessibility, financial affordability, and risk identification mechanisms in the governance of health equity. To address the persistent global challenges of health opportunity inequality arising from unequal resource distribution and socioeconomic disparities, the following internationally applicable policy recommendations and intervention pathways are proposed:

First, establish a precise resource allocation system to enhance the spatial accessibility of high-technology medical services. The findings indicate that surgical level is the primary determinant of health opportunity inequality, with a greater contribution among populations in better health, reflecting a structural trend in which “improvement in health is accompanied by increasing inequality in health opportunity.” Countries should develop a Disease–Resource Alignment Index (DRAI), based on disease burden data and regional health needs, to optimize the efficiency and spatial layout of advanced surgical resources. In addition, the Norwegian “Mobile Stroke Unit” model (28) may serve as a useful reference for improving temporal accessibility in resource-constrained regions through 5G-enabled remote surgical guidance and cross-regional collaboration platforms. The establishment of regional joint consultation mechanisms and inter-institutional cooperation networks could further reduce systemic inequalities arising from disparities in technological capacity.

Second, strengthen primary healthcare and human resource systems to consolidate the service foundation for health equity. The results show that the density of doctors and hospital beds are key environmental determinants of health opportunity inequality, underscoring the importance of human and infrastructural resources in shaping patient care pathways. Governments should prioritize the development of primary healthcare systems and the redistribution of health human resources as central strategies for promoting health equity. Measures such as improving the physical conditions of primary health facilities, providing professional incentives, and implementing cross-regional medical personnel rotation schemes can help promote the downward diffusion of high-quality resources. At the international level, the establishment of regional healthcare capacity-sharing mechanisms and multinational health cooperation networks (such as the EU’s cross-border healthcare collaboration framework) can enhance service capacity in disadvantaged areas, thereby realizing a more robust and balanced tiered healthcare system.

Third, reform healthcare financing systems to reduce service opportunity inequality driven by economic disparities. This study finds that the variance in medical expenses is a key economic determinant of health opportunity inequality, with a particularly high contribution among middle- and upper-quantile groups, suggesting that differences in financial capacity exert structural effects on healthcare utilization. To mitigate this, health insurance systems should transition from “basic coverage” to “equitable protection.” Specifically, a Tiered Co-payment System could be introduced, setting an upper limit on out-of-pocket expenses for lower-quantile patients (for instance, not exceeding 10% of household income), while adopting value-based reimbursement mechanisms for higher-quantile groups to prevent the dual imbalance of over-utilization among the wealthy and under-utilization among the poor. Moreover, drawing on Germany’s disease fund model (29), a dedicated long-term insurance scheme for cardiovascular and cerebrovascular diseases could be established to cover costs related to rehabilitation, nursing, and chronic disease management, thereby alleviating long-term financial burdens.

Fourth, advance prevention-oriented and data-driven health governance to shift the focus of health management upstream. Health opportunity inequality is shaped not only by access to treatment but also by access to prevention. Countries should therefore transition from a “disease-compensation” model toward an “opportunity-prepositioned” approach, developing public health policy frameworks centered on health promotion. This includes the establishment of a Digital Twin Early-warning System, integrating electronic health records (EHRs) with satellite remote sensing and environmental monitoring data to identify high-risk communities and enable proactive resource deployment. Furthermore, health literacy assessment should be incorporated into primary healthcare systems, while AI-assisted health education and behavioral interventions can enhance patients’ self-management capacities, thereby promoting health opportunity equality at the individual level. Such data-driven preventive interventions are applicable not only to low- and middle-income countries but also offer forward-looking insights for addressing health inequalities in high-income settings.

However, there are some limitations in this study. First, sample and regional limitations. The study primarily uses data from the middle-aged and older adults population in China, particularly focusing on cardiovascular and cerebrovascular disease patients. Therefore, the generalizability of the results may be constrained by regional and sample group limitations. Given the unique nature of China’s healthcare and social security systems, the applicability of the findings to other countries or regions requires further validation, especially in contexts with differences in economic conditions, healthcare resource distribution, and cultural backgrounds. Second, the availability and representativeness of data. This study relies on specific administrative data and hospital records, which, despite being anonymized and de-identified, still have limitations in terms of representativeness and comprehensiveness. Third, limitations of machine learning methods. While machine learning methods enhance the robustness of the study, they can lead to overfitting, causing the model to rely too heavily on training data, which may affect its generalizability in practical applications.

### Limitations

This study has several limitations that should be acknowledged. First, there are sample and regional limitations. The study primarily uses data from the middle-aged and older adults population in China, focusing on patients diagnosed with cardiovascular and cerebrovascular diseases. Therefore, the generalizability of the results may be constrained by regional and population-specific characteristics. Given the unique institutional features of China’s healthcare and social security systems, the applicability of the findings to other countries or regions requires further validation, especially in contexts with different economic conditions, healthcare resource distributions, and cultural settings.

Second, the availability and representativeness of data. The study relies on specific administrative datasets and hospital medical records, which, despite being anonymized and de-identified, still have limitations in terms of representativeness and completeness.

Third, the Shapley value decomposition was implemented using the TreeSHAP algorithm under the assumption of feature independence. While this assumption substantially improves computational efficiency and interpretability, it may overlook correlations among environmental variables—such as between healthcare resource density, institutional quality, and socioeconomic indicators. As a result, the estimated marginal contributions of some correlated variables might be partially confounded. Future work could employ conditional Shapley value methods (30) or other dependence-aware attribution frameworks to refine decomposition results under correlated feature settings.

Fourth, the transformation of the ordinal discharge diagnosis variable into a continuous scale using the rank-based inverse normal transformation (RINT) does not ensure equal distances between adjacent health categories (e.g., between “cured” and “improved” or between “not cured” and “death”). This limitation arises from the unknown distribution of the underlying latent health variable. Although the approach preserves ordinal information and allows for continuous modeling, the estimated health distances remain approximate. Future studies could explore more advanced methods to estimate inter-category spacing, thereby improving the precision of health inequality measurement.

Fifth, the analysis assumes the exogeneity of environmental variables. However, some factors—such as the surgical level received by patients—may be influenced by unobserved “effort-related” characteristics (e.g., health literacy, treatment-seeking behavior) or incompletely captured socioeconomic status. This potential endogeneity may lead to biased estimates of the true effects of environmental factors. Future research could consider instrumental variable approaches, natural experiments, or structural models to better identify causal relationships and isolate the exogenous component of environmental influences.

## Data Availability

The datasets presented in this article are not readily available because this study is based on administrative data from the medical insurance settlement platform of H Province, China. Requests to access the datasets should be directed to nuonuobiya@163.com.

## Footnotes

1
http://ghdx.healthdata.org/gbd-results-tool
